# The Interactions of Tau, RNA, and Stress Granules in Neurodegenerative Disease: A Comprehensive Review

**DOI:** 10.3390/cells15151365

**Published:** 2026-07-29

**Authors:** Tristyn N. Garza, Jose F. Abisambra

**Affiliations:** 1 Center for Translational Research in Neurodegenerative Disease (CTRND), University of Florida, Gainesville, FL 32610, USA; t.garza@ufl.edu; 2 Department of Neuroscience, University of Florida, Gainesville, FL 32610, USA; 3 Evelyn F. and William L. McKnight Brain Institute, University of Florida, Gainesville, FL 32610, USA; 4 Brain Injury, Rehabilitation, and Neuroresilience (BRAIN) Center, University of Florida, Gainesville, FL 32610, USA; 5 Genetics Institute, University of Florida, Gainesville, FL 32610, USA; 6 Brain Rehabilitation Research Center, Malcom Randall VA Medical Center, North Florida/South Georgia Veterans Health System, Gainesville, FL 32608, USA

**Keywords:** tau, RNA, stress granules, neurodegeneration, neurodegenerative disease

## Abstract

The discovery of RNA in the late 19th century revolutionized the understanding of cell biology. Subsequent discoveries over the next six decades revealed a key role for RNA in protein synthesis. Nevertheless, today, the mechanisms driving RNA metabolism remain enigmatic. Given its fundamental cellular role, RNA alterations are strongly linked to disease, including devastating neurodegenerative disorders pathologically defined by the accumulation of RNA-binding proteins. For example, the mislocalization of TDP-43, an RNA-binding protein, is a pathological feature of amyotrophic lateral sclerosis and frontotemporal dementia TDP-43. Another group of more than 20 neurodegenerative disorders, called tauopathies, is characterized by the aberrant accumulation of the protein tau. Similarly, the emerging concept that tau binds RNA, facilitating the formation of pathological structures, highlights the importance of RNA stability in tauopathies. However, the dynamics and consequences of RNA–tau interactions remain unclear. This review comprehensively catalogs key findings linking tau, RNA, and stress granules. These findings are important because they could offer novel opportunities to design therapeutic strategies.

## 1. Introduction

Cells require constant transcription and translation to maintain function. Given their unique role in brain connectivity, neurons need carefully regulated protein synthesis to perform optimally. Neuronal homeostasis and function demand accurate and efficient transcription, optimal ribosome biogenesis and assembly, and strict regulation of RNA translation.

During periods of stress, cellular adaptive pathways selectively regulate RNA translation. One example is by the formation of stress granules [1,2]. These membraneless complexes of protein, RNA, and translation factors serve many functions, including biomolecule storage, scaffolds for RNA-binding proteins, cell signaling, and the inhibition of translation [1,2,3]. In the early stages of Alzheimer’s disease (AD) and related disorders (ADRD), translation is impaired [4,5]. Although stress granules colocalize with pathological proteins associated with several neurodegenerative diseases, the mechanisms driving these translational deficits in disease remain elusive.

The microtubule-binding protein tau (MAPT) is expressed primarily in the central nervous system but can be found in the pancreas, muscle fibers, smooth muscle cells, and kidneys and serves a key role in neuronal function by stabilizing microtubules [6,7,8,9,10]. However, tau adopts toxic conformations under various stressors, culminating in the intracellular aggregation of tangles. Pathologically, AD is characterized by the appearance of tau tangles and amyloid-β plaques [11,12]. Clinically, patients present with impaired memory, struggle with reasoning and decision-making, experience difficulties speaking, an inability to perform normal daily tasks, and changes in personality, leading to social withdrawal [13]. In 2020, over 55 million people worldwide were diagnosed with AD, and, in 2025, approximately 7.2 million people were living with AD in the United States alone [13,14].

Studies identifying the tau interactome show that tau associates with numerous proteins, suggesting that it has many roles beyond microtubule stabilization. For example, Meier et al. [15] established that, in the endoplasmic reticulum within human brain cells, tau associates with many proteins associated with translation, with an increased association in AD brains. These results were later expanded using laser-capture and proteomics experiments, validating the importance of their findings. In conjunction with these non-canonical interactions, the association of tau and translational machinery correlates with altered translation in multiple models [15,16]. Interestingly, experiments using cell-free models show that oligomeric tau impairs the rate of translation, while synuclein oligomers (also associated with neurodegenerative diseases) do not [15]. Pathological tau associates with several RNA-binding proteins (RBPs) and ribosomal proteins (rp) in AD [15,16,17,18]. In cases of the accumulated dipeptide repeat expansion of C9ORF72, another pathological protein associated with amyotrophic lateral sclerosis (ALS) and frontotemporal dementia (FTD), structural and functional evidence shows profound ribosome dysfunction [19,20,21,22]. It is unclear if the interactions of tau with RNA and RBPs are direct or indirect; moreover, what drives these interactions and the consequences of their association remain poorly understood. They may present key insights into the pathogenesis and progression of tauopathies. In experimental models of tauopathy, the global shift in the translation of specific proteins, such that adaptive proteins are enriched and proteins involved in specialized functions (e.g., synaptic proteins) are decreased, suggests that there is a maladaptive response to stress. Stress granules (SGs) are another common feature of neurodegeneration [18,23,24]. These are typically transient structures that form under stressors such as heat, oxidative, and endoplasmic reticulum stress [1,3,25]. However, under periods of chronic stress, granules stabilize and sequester RNAs, RBPs, and ribosomal subunits [3]. In AD, pathological tau aggregates are associated with SGs; however, it remains unknown if tau aggregation precedes SG formation or vice versa [23,24]. This timeline is important to define causality and improve therapeutic targets.

While each of these components continues to be studied and reviewed individually, there are limited efforts integrating translation, stress, and neurodegenerative disease into a single focus. This review will highlight the complexity of translation, the effects of cellular stress, and tau biology in the context of neurodegenerative diseases.

## 2. Tau

**Figure 1:**
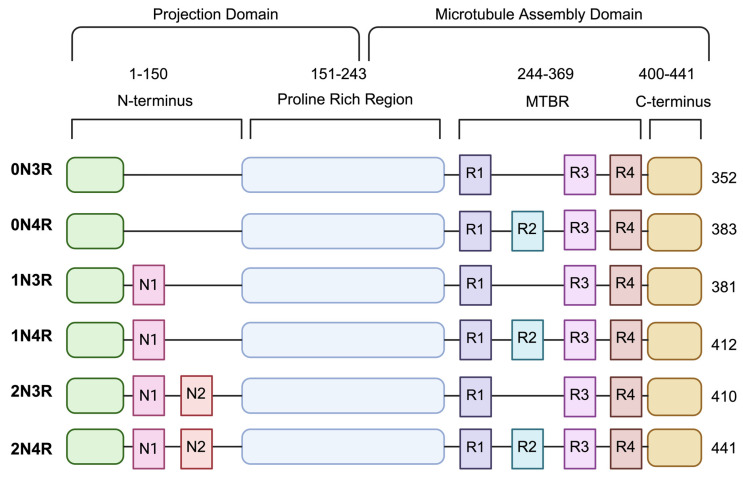
Tau isoform schematic, in ascending order of N-terminal inserts with 3R and 4R isoforms, including length. The figure highlights the N-terminal region, proline-rich region (PRR), microtubule-binding region (MTBR), and C-terminal region. Additionally, the projection domain and microtubule-binding domain are shown. Created in BioRender. Garza, T. (2026) https://BioRender.com/jbesod3 (accessed on 27 April 2026).

Tau is an intrinsically disordered protein, best known for the stabilization of microtubules in the central nervous system; however, recent data suggests that it participates in numerous other processes [10]. Tau is encoded on chromosome 17q21 by *MAPT* and composed of four regions, including an N-terminus (amino acids 1–150), proline-rich region (PRR; amino acids 151–243), microtubule-binding region (MTBR; amino acid 244–369), and C-terminus (amino acids 370–441) (Figure 1) [26,27,28]. Due to alternative splicing patterns, tau contains either three or four repeats of the MTBR (3R or 4R) and between zero and two repeats of the N-terminal inserts (0N, 1N, or 2N), resulting in tau isoforms that vary between 352 and 441 amino acids long (Figure 1) [29,30]. These splicing patterns include or exclude exons 2, 3, and 10, which encode the amino-terminal segments (exons 2 and 3) and one MTBR (exon 10) [31]. The adult human brain normally expresses both 3R and 4R tau isoforms in equal proportion. However, the balance of 3R and 4R tau varies with age; specifically, fetal tau primarily consists of 3R isoforms, and, as humans age, the 3R/4R ratio evens [32].

The longest form of tau (2N4R) has an overall basic composition with roughly the same amount of positive, negative, and aromatic residues. However, the amino-terminal (N-) region is acidic, and the carboxy-terminal (C-) region is neutral [33]. Based on tau interactions with microtubules, the C-terminus is defined as the assembly domain, and the N-terminus is the projection domain guiding protein interactions [27]. The N-terminus also contributes to tau localization in axons [34].

### 2.1. Non-Canonical Tau Functions: Beyond Microtubules

Recent studies suggest that tau has other functions beyond microtubule stabilization. ER stress is a common feature in neurodegenerative diseases, and, in 2013, interactions between pathological tau and the ER were identified [35]. Increased tau promotes the unfolded protein response, a stress pathway that activates protein kinase RNA-like ER kinase (PERK) to reduce the translation of proteins entering the ER, allowing other mechanisms to resolve the stress event and ER overloading. In 2015, 92 tau-associated ER proteins were identified, 77 of which had not been previously identified as associated with tau, and 39 were unique to the AD brain [36]. This study also identified rpL28 and rpLP0 as associated with tau, and these interactions were more robust in AD brains [36]. In addition, tau-associated RNA-binding proteins, specifically ribosomal proteins, were identified [36]. These data revealed numerous other pathways and functions involving tau in the context of disease.

Neurofibrillary tangles (NFTs) include 75 tau-interacting proteins that are associated strongly with phosphorylated tau species [37]. In addition, phosphorylated tau aggregates interacted more robustly with neuron-specific proteins compared to non-phosphorylated tau. The most prominent enrichment was found in the two main degradation systems: the ubiquitin–proteosome and lysosome systems [37]. In 2022, proteomic analysis was used to elucidate the tau interactome in iPSC-derived neurons (WTC11) with a doxycycline-inducible transgene to express 2N4R tau with an APEX2 flag-tag to insert P301L and V337M mutations through cloning [38]. This study found that tau has distinct interactions between the N-terminal and C-terminal regions and that tau directly interacts with proteins involved in mitochondrial bioenergetics such as SLC25A4, an ATP/ADP transporter [38]. Together, these studies suggest that tau also participates in protein degradation and mitochondrial function.

Tau also interacts with spliceosome components in AD brains, and, in *Drosophila*, changes in the spliceosome promote tau toxicity [39]. When human tau was spliced into *Drosophila*, tau reduced spliceosome components, which led to loss of function in the core splicing protein, SmB, resulting in more cryptic splicing events [39]. Therefore, tau interactions with mRNA, specifically the spliceosome, disrupt small nuclear ribonucleoprotein (snRNP) function [39].

### 2.2. Tau Modifications

Tau can undergo several types of post-translational modifications (PTMs) on approximately 35% of its amino acids, specifically cysteine, histidine, asparagine, arginine, lysine, tyrosine, threonine, and serine [40]. Potential PTMs include glycosylation, nitration, glycation, ubiquitination, and phosphorylation [12,41]. In relation to AD, tau hyperphosphorylation and aggregation is a key pathological feature [25,42,43,44,45]. Under normal conditions, tau does not typically aggregate; however, hyperphosphorylated tau is enriched in paired helical filaments (PHF), first described by Kidd [46], or NFTs, first described by Alzheimer [11]. The aggregation of tau in PHFs or NFTs is associated with many neurodegenerative disorders, which include AD, Pick’s disease, Huntington’s disease, progressive supranuclear palsy (PSP), corticobasal degeneration (CBD), and frontotemporal dementia with parkinsonism-17 (FTDP-17) [27,43].

Tau phosphorylation is an important physiological regulator of binding affinity, and it is required for the stabilization of microtubules [27,47,48,49,50]. As tau becomes hyperphosphorylated, it dissociates from microtubules and misfolds, gaining affinity for other tau monomers, and aggregates into tau oligomers [51,52]. Tau oligomers precede the formation of PHFs and NFTs, and these intermediate oligomers can be soluble or insoluble, have an alternative conformation, and are formed early in tau aggregation [53]. Multiple studies show that tau oligomers contribute to seeding, spreading, tangle formation, neuronal toxicity, and impaired memory consolidation [51,52,53].

In addition to PTMs, over 60 MAPT mutations are associated with neurodegenerative disease. For example, P301L is a point-missense mutation in exon 10 where codon CCG (proline) is converted to CTG (leucine). Because this mutation is contained in exon 10, it is only found in 4R tau [54,55,56,57]. The P301L mutation is associated with FTD, promotes β-sheet folding, increases PHF formation, and reduces tau-mediated microtubule stabilization [54,55,56,57]. Another mutation of interest is P301S, similar to P301L, this point-missense mutation converts CCG (proline) to TCG (serine), is associated with FTD, and reduces tau-mediated microtubule stabilization [58,59]. ΔK280 is unique as a deletion of AAG (lysine) in exon 10 and is associated with the risk of multiple diseases, including AD, FTD, and Pick’s disease (60–61). Although ΔK280 is on exon 10 (only 4R tau), it leads to abundant 3R tau, as ΔK280 inhibits the inclusion of exon 10 through splicing, thus reducing microtubule assembly [60,61]. Moreover, another mutation of interest is S320F, a point-missense mutation of TCC (serine) to TTC (phenylalanine) on exon 11 [62]. S320F is associated with FTD and Pick’s disease, reducing microtubule formation and inducing the loss of a phosphorylation site within tau [62]. Transgenic models overexpressing these mutations have enriched the field and provided critical information about tau biology. Recently developed models combining P301L and S320F expression highlight that overexpression promotes aggressive deposition, providing key insights into aggregation dynamics [63]. Knock-in models expressing these two mutations (and a third mutation), promoting the retention of exon 10, showed aggressive pathology and cognitive deficits within 6 months [64].

### 2.3. Tau in Neurodegeneration

AD is pathologically characterized by amyloid-beta plaques (Aβ-plaques) and hyperphosphorylated tau in NFTs [11,12]. Although tau is important for optimal neuronal function, toxic conformations and aggregates in brain cells contribute or lead to tauopathies. These include oligomers, PHFs, and NFTs, forming unique structures in different cell types, such as tufted astrocytes in PSP [52]. Tauopathies are also classified as primary or secondary; in primary tauopathies, the deposition of pathological tau precedes other pathogenic drivers of neurodegeneration, whereas, in secondary tauopathies, other promoters of toxicity (e.g., pathological amyloid development in AD and Down syndrome) precede tau pathology [65].

In AD, there is the progressive spread of tau pathology throughout the brain. The extent of pathological tau distribution is classified in terms of Braak staging (I–VI) [66,67]. Pathological tau aggregates first appear in the transentorhinal region (I) and expand into the entorhinal region (II), and then the neocortex of fusiform and lingual gyri (III). From the neocortex, tau pathology spreads to the mature neocortex and associated areas (IV); then, the pathology propagates to the frontal, parietal, and occipital lobes (V). Finally, the pathology is evident throughout the brain (VI) [66].

In 2016, a more thorough AD classification was established based on the presence of amyloid deposits (A), tau tangle aggregates (T), and neurodegeneration (N) evidenced by neuroimaging [68]. The A/T/N system also has six distinct stages, where patients are asymptomatic in stages 1 and 2. In stages 3, 4, 5, and 6, patients exhibit early, mild, moderate, and severe AD dementia, respectively [68]. Given that the prevailing mechanistic model of AD is the amyloid cascade hypothesis, which posits that amyloid deposition precedes tau pathology, AD is considered a secondary tauopathy [69]. In contrast, primary age-related tauopathy (PART) is a neurodegenerative condition characterized by tau burden (NFTs) that peaks at Braak IV without Aβ plaques [70]. These NFTs are found primarily in the temporal lobe and hippocampus and do not progress to other brain regions. Importantly, NFTs are common in aged brains and have been found in younger individuals; however, the pathological composition of PART aligns with mild cognitive impairment akin to that in AD Braak IV patients [70].

PSP is a primary 4R tauopathy and is pathologically identified by globose-type NFTs, tuft-shaped astrocytes, and compromised oligodendrocyte integrity [52]. Patients with PSP have increased levels of total tau, phosphorylated tau, NFTs, and tau oligomers. These oligomers are found in glial cells and neurons, suggesting a role for tau oligomers in pathology prior to the formation of NFTs [52]. Phosphorylation plays a large role in tau aggregation and toxicity, and it was recently determined from AD and PSP tissue that glutamate-substituted S305 in the MTBR, in repeats R1-R2 specifically, changed the tau seeding activity in AD but not PSP [71]. Moreover, the addition of phospho-mimicking substitutes in the C-terminal reduced tau seeding in both AD and PSP, but phospho-mimicking substitutions in the PRR did not alter seeding in either AD or PSP [71].

Under the umbrella of FTD, neurodegenerative diseases can be 3R, 4R, or combination 3R/4R [72,73]. Across 38 patients with frontotemporal lobar degeneration tau mutations (FTLD-MAPT), an increased tau burden in the grey matter was associated with increased neurodegeneration [73]. The severity of the tau burden in the grey matter varied based on the tau isoforms, where 3R had a lower burden than 4R, but 3R and combination 3R/4R resulted in increased severity of neurodegeneration [73]. The tau isoform also influenced where the most severe pathology was observed: 4R tau (P301L) had the most severe neuronal pathology and the second-most severe glial pathology, whereas 3R/4R (L315R, L266V, R406W) had the most severe glial pathology [73].

In chronic traumatic encephalopathy (CTE), the onset of which is strongly associated with mild repetitive traumatic brain injury (TBI), the brain also exhibits tau pathology [74,75,76]. In fact, repetitive mild TBI (rmTBI) increases the risk of developing numerous neurodegenerative diseases, and many of them feature tau pathology [76]. Although the cryo-EM structures of tau in AD and CTE share common folding patterns, recent data suggest that there are broader differences in tau phospho-epitopes. Surprisingly, exposing C57BL/6J WT tau mice to rmTBI led to decreased levels of pS396 tau in the optic tract but increased pS199, suggesting unique TBI-associated pathogenic tau cascades [76]. Repetitive TBI is strongly associated with an increased risk for CTE, which is diagnosed post-mortem through a distinct pattern of tau accumulation [74,77]. Specifically, tau in CTE is irregularly distributed, and it appears as hyperphosphorylated aggregates in neurons and astroglia at the cortical sulci [77]. Walker et al. [74] reviewed several TBI studies focused on mild to severe TBI (msTBI) progressing to CTE and found that msTBI can contribute to long-term tau pathology. The complex and multifactorial nature of head injuries creates a complicated panorama in which to predict outcomes. Age, for example, is a powerful confounding factor in recovery after injury, and this is likely because of reduced cognitive resilience [78]. Moreover, contributors to other tauopathies, such as the expression of hypomorphic PERK, which increases the risk for PSP, or the overexpression of FTD-associated mutant tau (P301L), worsen outcomes [75,79]. Interestingly, WT tau mice exposed to rmTBI did not show overt phospho-tau reactivity; in contrast, the overexpression of P301S tau led to increased AT8 reactivity in the motor and sensory cortices after rmTBI [75]. Moreover, tau was mislocalized to the dendrites of P301S rmTBI mice [75].

In Parkinson’s disease, pathological tau often appears as a secondary pathology to alpha-synuclein aggregation into Lewy bodies [80]. Similarly, ALS brains often present tau aggregates secondary to TDP-43 co-pathology. Tau aggregation is also a secondary pathology in Huntington’s disease [81], and NFTs are often found in Huntington’s patients with aggregation and pathological spread similar to AD [82].

Tau pathology also affects peripheral tissues. Many clinical studies show that populations with tauopathies have muscle weakness, and several mouse models exhibit motor and muscle abnormalities [83,84,85]. In humans, decreased grip strength is associated with cognitive impairment and progression to AD [83,84]. In mice, brain P301L expression coincides with decreased grip strength and sex-specific transcriptional changes in the myofibers proximal to blood vessels and nerve bundles [85]. Interestingly, muscle alterations were intrinsic to the myofibers and independent of motoneuron function, suggesting that the impact of brain P301L tau expression on muscle is not dependent on neuronal connectivity.

Finally, a larger tau variant called “big tau”, which is primarily found in the periphery, results from the retention of an exon typically spliced out in the brain. While it is not directly associated with primary or secondary tauopathies, big tau aggregates appear in the peripheral vasculature under disease conditions [86].

## 3. RNA

### 3.1. Different RNAs and Their Functions

RNA chains adopt many structures and perform multiple functions, and they primarily exist as messenger RNA (mRNA), transfer RNA (tRNA), and ribosomal RNA (rRNA) [87,88,89,90]. Other types of non-coding RNAs (ncRNAs) include non-coding small nuclear RNA (snRNA) and small nucleolar RNA (snoRNA), which are responsible for RNA splicing and modification; micro-RNA (miRNA), involved in the regulation of mRNA levels; double-stranded non-coding small interfering RNA (siRNA), named for its interference in translation and gene expression; and long non-coding RNA (lncRNA) and circular RNA (circRNA), which function as gene expression regulators [90,91,92,93,94,95,96,97,98,99,100,101,102,103]. There are also many RBPs that guide the RNA life cycle, including regulation, export, localization, stability, and protein–protein interactions [104].

Messenger RNA is translated within ribosomes in conjunction with tRNA, which is recognized by eukaryotic elongation factor 1α (eEF1α), where nucleotide triplets (codons) move through the A (acceptor) site to bind the tRNA anticodon or P (peptidyl) site, elongating the polypeptide chain, and the E (exit) site to be released by the ribosome [105,106,107,108]. The first two nucleotides in a codon follow a strict Watson–Crick pairing, but the third nucleotide can have irregular pairing, called ‘wobble pairing’ [102]. When uridine is in the wobble position of the anticodon (U_34_), it can be altered via two enzymatic methods, via the elongator complex or cytosolic thiouridylase subunits 1 and 2, both enhancing the decoding process of AAA, CAA, and GAA [109]. This tRNA–uridine modification works in conjunction with kinase mechanistic target of rapamycin complex 1 (mTORC1) to maintain the synthesis of ribosomal proteins [109]. mTORC1 is part of the mammalian target of rapamycin (mTOR) family, and it is a key regulator of protein synthesis; specifically, mTORC1 phosphorylates both eIF4E-binding proteins and ribosomal protein small 6 kinase 1 (S6K1), promoting mRNA initiation and elongation [109,110,111]. When tRNA is not modified in the wobble position by uridine, mRNA builds up and ribosomal protein production decreases as most ribosomal proteins have high AAA content [109].

Additionally, tRNA can be altered in other ways, including post-translational modification, mutations in tRNA genes, or by enzymes like U_34_, as mentioned earlier. While enzymatic changes like U_34_ facilitate tRNA function, mutations on tRNA can be detrimental. For example, mitochondrial tRNA is encoded by one gene, and mutations can lead to several diseases, such as mitochondrial encephalomyopathy, lactic acidosis, and stroke-like episodes (MELAS) and myoclonic epilepsy with ragged red fibers (MERRF) [106,107]. Conversely, nuclear tRNA is encoded by redundant genes, but disease mutations alter selenocysteine incorporation, leading to familial dysautonomia, intellectual disability, ALS, Galloway–Mowat syndrome, and early-onset epileptic encephalopathy [106,107,112].

### 3.2. RNA Interactions with Tau

Tau is an RNA-binding protein; it interacts with rRNA, tRNA, mRNA, and single-stranded and double-stranded RNA [113,114]. In *E. coli*, tau’s ability to interact with RNA is dependent on the presence of both the MTBR and PRR; the removal of either region results in the loss of tau–RNA interaction [114]. Due to RNA being negatively charged, RNA neutralizes the positive residues of tau, promoting the aggregation of tau into PHFs [34,113,114,115].

RNA–tau interactions were confirmed in human embryonic kidney (HEK) cells expressing full-length wildtype tau (2N4R), P301L tau (mutant associated with FTD) (2N4R), as well as human-induced pluripotent stem cell-derived neurons (iPSC neurons) [88]. Previously, Kampers et al. [113] highlighted that tau rapidly formed filaments in the presence of tRNA, and, in 2017, crosslinking and immunoprecipitation identified that most tau–RNA interactions were primarily tRNA–tau in HEK-WT, HEK-P301L, and iPSC neurons [113,116]. In both HEK and iPSC cell models, tau was crosslinked with tRNA primarily in the anticodon loop, with less interaction in the T-loop and D-loop [116]. In addition, as tau and RNA interact, they condense into droplets (coacervates), and, over time, they adopt a pathological conformation within the droplet [116].

While tau preferentially associates with tRNA, several other tau–RNA interactions have been identified. In tissue from the dentate gyrus (DG) and CA1 of the hippocampus in patients with AD (Braak stages I–VI and middle-aged patients without pathology) show that the mRNA of nucleolar genes was decreased in later stages of AD, specifically Braak stages V–VI (Braak stage I–II: low tau burden, Braak stage III–IV: moderate tau burden, Braak stage V–VI: severe tau burden) [66,67,117]. However, when comparing the rRNA of controls to AD, the 28S rRNA of AD tissue was downregulated in the DG and CA1 in Braak stages III–IV; conversely, in Braak stages V–VI, 28S rRNA was upregulated [89]. However, 18S rRNA was not altered between AD and controls or between areas of the hippocampus [117]. Furthermore, full-length (2N4R) tau fibrils from AD tissue were bound primarily to 18S rRNA, as well as mRNA, mitochondrial RNA, and miRNA [118]. Surprisingly, while multiple species of RNA bind to AD tau, recombinant tau (tau40) only associates with 18S rRNA, forming paired fibrils [118]. Both recombinant tau–18S rRNA and AD tau–18S rRNA form C-terminal cores; however, in AD, the core forms in residues 341–441, while the recombinant tau core forms in residues 273–341 of the MTBR in repeats 2 and 3 [118]. In recombinant tau40 and total RNA, the C-terminal core is found in residues 391–426 [119]. Each tau–RNA interaction forms unique fibrils, with varying degrees of stability, suggesting the RNA binding of tau as an important component of tau aggregation. These studies highlight tau–tRNA and tau–rRNA interactions, but tau also has many interactions with mRNA.

Tau–mRNA interactions influence transcription through alternative poly-adenylation pathways, association with the spliceosome, and nuclear chromatin [120]. Specifically, using tau-expressing HEK models, WT tau upregulated cytoskeletal gene expression and downregulated Golgi and mitochondrial gene expression compared to HEK-P301L tau [120]. Whereas, HEK-P301L upregulated gene expression related to axonal microtubules and ribonucleoproteins [120]. Interestingly, tau associated with mRNA is often found in ribonucleoprotein (RNP) structures such as stress granules [18,23,121]. Furthermore, poly(A)-RNA bound to tau promotes tau oligomerization [122]. In fact, tau bound to poly(A)-RNA more readily than tubulin, disrupting microtubule assembly [122]. Although poly(U)- and poly(C)-RNA also bind to tau, only poly(A)-RNA can induce fibril formation [123]. Beyond these extensive tau–RNA interactions, tau is capable of a multitude of protein–protein interactions.

### 3.3. RBP Interactions with Tau

Tau also interacts with many RBPs [15,17,18,23,24,36,120,124]. Experiments in human-derived iPSC neurons show that tau interacts with RNA-binding proteins, but specific mutations TauV337M and TauP301L weaken the interactions with some ribosomal proteins [38]. RBPs Musashi1 and Musashi2 (MSI1 and MSI2, respectively) are evolutionarily conserved, participate in the cell division of neuronal precursor cells, and interact with tau oligomers in AD brains [125]. Not only does tau colocalize with MSI1 and MSI2 in the cytoplasm and nucleus, but tau and MSI proteins both promote tau–MSI nuclear localization and accumulation [17]. The tau–MSI aggregates destabilize nuclei by altering nuclear transport via Importin-β and LaminB1, fostering tau accumulation in the nucleus [17]. These are only a few of the RBP–tau interactions associated with neurodegenerative disease.

It is important to note that RBPs are also implicated in neurodegenerative diseases without tau. Transactive response DNA-binding protein 43 (TDP-43) is an RBP with roles in RNA splicing, mRNA transport, and pre-microRNA processing, and heterogeneous nuclear ribonucleoprotein A1 (hnRNPA1) is involved in pre-mRNA splicing, nuclear–cytoplasmic shuttling, and translation [126]. Similar to tau, TDP-43 is associated with neurodegenerative diseases, specifically ALS, FTD, and FTLD, and forms pathological aggregates [127]. Tau and TDP-43 can be transported through the secretome or extracellular vesicles or as free proteins [127]. TDP-43 has its own implications in neurodegeneration; in iPSC neurons, when TDP-43 and hnRNPA1 are knocked down, translation and mRNA transport in the neuron are affected [128]. In neurites, transcripts are heavily enriched and translated locally. More specifically, TDP-43 knockdown increases translation in neurites with decreased neurite outgrowth, whereas hnRNPA1 knockdown minimally impacts mRNA transcript levels but hnRNP3 increases to compensate and maintain proteostasis [128]. In 5xFAD mice (five familial AD mutations), APP/PS1 mice, AD APPswe cells, and AD patient serum, RNA sequencing showed the upregulation of lncRNA growth arrest-specific 5 (GAS5). GAS5 is expressed in the cerebral cortex and involved in cell proliferation, vascular remodeling, and apoptosis. GAS5 contributes to AD pathology by increasing cognitive dysfunction, tau phosphorylation, and Aβ production [129]. TDP-43 also interacts with tau, colocalizing with NFTs in AD and exacerbating tau pathology [130]. When together, tau–TDP-43 form irregularly shaped condensates, promoting TDP-43 aggregation, while suppressing tau fibril formation but increasing oligomeric tau and tau–TDP-43 assemblies [130]. Taken together, tau interacts with many RNAs and RBPs within the cell, altering both the translational and transcriptional landscape, which may induce cellular stress and lead to neurodegeneration.

## 4. Stress Granules

A major response to cellular stress is the formation of stress granules (SGs). These temporary organelles are membraneless assemblies of RNPs, a variety of RNAs, including mRNA and non-coding RNA, RBPs, translation initiation factors, ribosomal components, and poly(A) sequences [3,25,131,132,133]. While SGs were first described in plants, both their formation and function are highly conserved across species, including *Aradopsis*, yeasts, *C. elegans*, and mammals [1,131,132]. SGs are composed of a core structure surrounded by a dynamic shell that exhibits liquid–liquid phase separation (LLPS), enabling protein–protein interactions [3]. Two major pathways trigger SG formation: either the phosphorylation of eIF2α or the phosphorylation-independent pathway by eIF4A with the goal of releasing mRNA from polysomes (multiple ribosomes on a single mRNA transcript) [25]. However, the formation of SGs can be triggered by a variety of stressors, including heat, oxidation, and starvation, and each cue has a corresponding time required for SGs to form [1,25]. The inhibition of translation initiation promotes the formation of SGs to sequester RNA, upregulate the synthesis of specific stress response proteins, and promote cell signaling [1,132]. SGs are transient, and, when cellular stress resolves, SGs dissipate, allowing translation to resume [132,133]. However, there is aberrant accumulation of SGs and a continued reduction in translation during periods of chronic stress, such as viral infection, cancer, and neurodegenerative disease [25].

### 4.1. SGs and Translation

While ribosomal subunits are created at a 1:1 ratio (40S small subunit–60S large subunit), SGs have a higher concentration of 40S subunits than 60S and functional 80S monosomes [2]. The recruitment of 40S and 60S subunits to SGs is thermodynamically driven, and SGs protect the 40S subunits from decay during periods of stress [2]. Transcriptome analysis revealed that mammalian SGs are primarily composed of mRNA (specifically actin), with some ncRNA present; however, each SG will vary slightly in its composition [131]. Messenger RNA that is sequestered from polysomes is dynamic, such that the mRNA can coalesce in an SG and later transfer to a processing body (P-body) for degradation or return to the polysome to resume translation [133,134]. While it was believed that the only stalled mRNAs were recruited to SGs, live-cell imaging in conjunction with SunTag translation assays in HeLa cells show that mRNAs in active translation are recruited to SGs [135]. This suggests that the mechanism for translation suppression is not the SG itself, as mRNAs localized to SGs can be synthesized into proteins [135].

### 4.2. SGs and Tau

SGs also sequester kinases and phosphatases, linking SGs to MAPK signaling, nuclear factor-kB, p53, and mTOR pathways [132,136]. When the stressors are removed, the disassembly of SGs is guided by chaperones, microtubules, and autophagy, allowing the release of the stored mRNA and proteins [24]. SGs contain over 400 proteins, 252 of which are RBPs [1]. In periods of prolonged stress, SGs aggregate, becoming toxic, similarly to tau aggregation [124]. SGs can be transported within the cell via microtubules or actin [137]. Microtubules tend to be denser in the perinuclear region, whereas actin is denser in the periphery, and SGs have a high affinity for β-tubulin in microtubule-rich regions [132]. SGs are a common feature in neurodegenerative diseases, including ALS, FTD, AD, Huntington’s disease, and Creutzfeldt–Jakob disease [18,23,24]. Many SG markers, such as T-cell intracellular antigen-1 (TIA-1) and SG-associated RBPs, colocalize with pathological phosphorylated tau in the rTg4510 and PS19 (P301S tau) mouse models of tauopathy [18,23]. TIA-1 and tristetraprolin (TTP) are nucleation proteins for SGs, and the rate at which TIA-1 colocalizes with tau correlates with the size of the tau aggregate [23]. In AD and FTDP-17, as the disease progresses, the quantity of SGs increases, promoting colocalization with pathological tau aggregates [23]. The frequency of RBP–tau colocalization also changes over the course of disease [18]. As tau pathology increases, most tau–RBP association decreases; however, specific RBPs (associated with ALS) accumulate with progressing tau pathology, including EWSR1, TAF15, and hnRNPA0 [18]. SG formation is guided by LLPS for protein–protein interactions. Tau also experiences LLPS that is accelerated by mRNA, potentially creating a feedback loop for SG formation and tau aggregation [18].

Although tau interactions are the primary focus of this review, it is not the only neurodegenerative-associated protein to have SG interactions. Both TDP-43 and fused in sarcoma (FUS) are associated with persistent SG deposition [18]. Similarly to tau, disease-associated TDP-43 colocalizes with TIA-1, and FUS colocalizes with PABP-1 and ATAXIN2 [24,124]. TDP-43 is an RBP with roles in RNA splicing, mRNA transport, and pre-microRNA processing, and, when hyperphosphorylated or cleaved, TDP-43 becomes pathogenic, aggregates, is recruited to SGs, and associates with FTD-ALS [124,138]. FUS is involved in transcription, splicing, transport, and translation. In ALS, mutant FUS increases its recruitment to SGs [24,124,139]. In addition, SGs also play a role in polyglutamine (polyQ) diseases like Huntington’s disease [1]. PolyQ diseases involve the abnormal expansion of CAG repeats in the open reading frame, which assemble on each other and aggregate [1]. These polyQ aggregates sequester RBPs commonly found in SGs and colocalize with TIA-1, although this is not as well defined [1]. While TDP-43 and FUS have better-defined interactions with SGs than tau, the pathological aggregation of both RBPs can be found in tauopathies. This suggests that SG formation, accumulation, and interaction may be downstream effects of pathological tau aggregation.

## 5. Discussion

Tau interacts with mRNA, tRNA, and rRNA, but we do not yet know the driving factors or the molecular consequence of these interactions or if there is a preference for a specific species of RNA. SGs are a common feature of several neurodegenerative diseases, and they are abundant in tauopathies. Under stress, the production of synaptic proteins may be of lower priority, and our previous work suggests that tau arrests the translation of synaptic proteins. Although the formation and function of SGs is well established, and tau aggregation triggers stress responses, it remains to be determined if tau–RNA and tau–RBP interactions can also induce SG formation. Many studies have investigated tau–RNA associations, tau stress, and tau neurodegeneration, but research is yet to combine these interactions into a complete pathway. This work is ongoing and is expected to have a major impact in understanding tau biology and diseases of tau aggregation.

## 6. Conclusions

Tau was initially identified as a microtubule-stabilizing protein. However, this review highlights several studies showing that tau plays many roles across cellular processes and in neurodegenerative disease. While tau interactions and the translational mechanisms discussed are normal processes, these can become pathological and activate stress responses. In turn, the appearance of SGs often coincides with aberrant tau accumulation. During transient stress, SGs can dissociate; however, in chronic stress, as in neurodegenerative diseases, SGs do not disassemble. This can create a negative feedback loop where more SGs lead to the accumulation of tau, a continued decrease in translation, and increased translational errors. A major challenge is identifying which interactions occur first as this could reveal disease pathways and effective therapeutic targets. We highlight that tau interacts with multiple types of RNA, many RNA-binding proteins, and stress granules, but it is still unknown which interactions are part of normal cellular function and what drives pathology. While many mechanisms still need to be studied, this review presents many potential pathways by which to answer the fundamental question of how tau interactions influence translation in neurodegenerative diseases.

## Data Availability

No new data were created or analyzed in this study. Data sharing is not applicable to this article.
